# Andean Berry (*Vaccinium meridionale* Swartz) Juice Promotes Cytotoxic and Proapoptotic Effects in Human Early-Stage and Metastatic Colorectal Cancer Cells

**DOI:** 10.3390/molecules31122147

**Published:** 2026-06-18

**Authors:** Ivan Luzardo-Ocampo, Myriam Agudelo-Quintero, Sandra S. Arango-Varela, Silvia A. Quijano, Maria E. Maldonado-Celis, Jorge A. Lopera-Rodríguez

**Affiliations:** 1 Tecnologico de Monterrey, Institute for Obesity Research, Ave. Eugenio Garza Sada 2501 Sur., Col., Tecnológico, Monterrey 64700, Nuevo Leon, Mexico; ivanluzardo@tec.mx; 2 Tecnologico de Monterrey, School of Engineering and Sciences, Ave. Eugenio Garza Sada 2501 Sur., Col., Tecnológico, Monterrey 64700, Nuevo Leon, Mexico; 3 Research and Graduate Program in Food Science, School of Chemistry, Universidad Autónoma de Querétaro, Cerro de las Campanas S/N, Querétaro 76000, Queretaro, Mexico; magudelo07@alumnos.uaq.mx; 4 Grupo de Investigación e Innovación Biomédica, Facultad de Ciencias Exactas y Aplicadas, Instituto Tecnológico Metropolitano (ITM), Calle 73 No. 76A-354 Vía al Volador, Medellín 050012, Antioquia, Colombia; alejandrolopera@itm.edu.co; 5 Grupo de Investigación Biología Médica, Facultad de Ciencias Exactas y Aplicadas, Instituto Tecnológico Metropolitano (ITM), Calle 73 No. 76A-354 Vía al Volador, Medellín 050012, Antioquia, Colombia; 6 Grupo de Investigación en Ecología y Conservación de la Biodiversidad (EcoBio), Facultad de Ciencias Básicas, Universidad Santiago de Cali, Cali 760001, Valle del Cauca, Colombia; 7 Escuela de Nutrición y Dietética, Universidad de Antioquia, Carrera 75 No. 65-87, Medellín 050034, Antioquia, Colombia; maria.maldonado@udea.edu.co

**Keywords:** Andean berry (*Vaccinium meridionale* Swartz) juice, apoptosis, colorectal cancer, antiproliferative activity, metastasis

## Abstract

Andean berry (*Vaccinium meridionale* Swartz) is an underutilized fruit that could serve as a source of bioactive compounds with biological properties associated with apoptosis and cytotoxicity in colorectal cancer cells. This study aimed to evaluate the cytotoxic and proapoptotic effects of Andean berry juice (ABJ) in human SW480 and SW620 colon cancer cell lines, which represent early-stage and metastatic colorectal cancer, respectively. The juice was prepared from freeze-dried fruits, and several concentrations were assayed in cells. Bioactive compounds in ABJ showed the strongest reductions in metabolic activity and proliferation observed in SW620 cells. ABJ treatments promoted early apoptosis while inducing cell cycle arrest in the S phase (SW480) and in the G2/M (SW620). Mild mitochondrial depolarization was observed, while increased reactive oxygen species (ROS) accumulation was detected in both cell lines. More proteins involved in the apoptotic process were modulated in SW620 cells, whereas SW480 displayed greater fold changes in regulatory and stress-response proteins. Proteomics and bioinformatics analyses suggested that extrinsic apoptosis predominated in SW480 cells, whereas intrinsic apoptosis was observed in SW620 cells. These results highlighted the cytotoxic and pro-apoptotic potential of the combined activity of polyphenolic compounds from ABJ, demonstrating distinct mechanisms in vitro.

## 1. Introduction

The development of cancer is a multistage process in which cells acquire characteristics, such as uncontrolled growth, evasion of tumor suppressor signals, and resistance to cell death, ultimately reaching a state of replicative immortality that promotes their transformation into malignant cells. These traits are considered important targets for the development of new cancer treatments [1]. Colorectal cancer (CRC) originates in the epithelium of the colon and/or rectum, and 90% of cases occur sporadically, without a family history, for which the underlying causes are largely unknown [2]. According to a recent report, colorectal cancer is the third most diagnosed cancer and the second leading cause of cancer-related death, accounting for 1.9 million new cases and nearly 1 million deaths in 2022 [3]. Other research estimates that by 2030, the number of cases will increase by 60%, reaching 2.2 million new diagnoses and 1.1 million deaths globally as a consequence of population aging [4,5].

The therapeutic management of CRC includes chemotherapy, radiotherapy, and surgical resection of the tumor, among other treatments [6]. Therapies targeting cancer-related genes have become especially important in metastatic CRC. These therapies, including the anti-epithelial growth factor receptor (EGFR) antibodies (e.g., cetuximab and panitumumab) and the vascular endothelial growth factor receptor (VEGFR) antibodies (e.g., bevacizumab, ramucirumab, and aflibercept), have become a critical component of treatment [7]. Other treatments include targeted epigenetic modifications, the use of DNA or histone demethylase inhibitors, or targeting specific metabolites, such as glucose, lipid, or glutamine metabolism [8].

Chemotherapeutic agents cause adverse effects, including damage to epithelial and liver cells, leading to symptoms such as fatigue, nausea, hair loss, and anemia [9]. These effects result from their nonspecific cytotoxic activity against both cancerous and healthy cells [10]. One approach that has been studied as a complementary strategy to chemotherapy is chemoprevention, which aims to harness the biological activity of phytochemicals present in foods and edible plants to prevent cancer development and progression [11]. Research in this field has shown that phytochemicals, such as phenolic compounds, can exert cytotoxic effects on cancer cells by interacting with molecules in cell signaling pathways associated with proliferation and by inducing cell death [12].

One group of foods that has been evaluated for its potential chemopreventive properties is berries, particularly species belonging to the *Vaccinium* genus. One such species, *Vaccinium meridionale* Swartz, commonly known as Andean berry, mortiño, or agraz, grows in Andean regions at elevations ranging from 2000 to 3000 m above sea level [13] and is used in processed products such as jams and juices. Several bioactive compounds have been identified in this species, including phenolic acids (e.g., chlorogenic and caffeic acid derivatives) and several types of flavonoids, such as the anthocyanins cyanidin-3-glucoside, cyanidin-3-galactoside, and delphinidin-3-glucoside [14,15]. Previous studies have shown that most phenolic compounds in the juice remain bioaccessible after gastrointestinal digestion [16,17].

Furthermore, our research group has contributed to the comprehensive characterization of Andean berry juice using chromatographic techniques and has identified several polyphenolic compounds consistent with previous reports. In addition, aqueous extracts of this berry demonstrated antiproliferative activity against the colon adenocarcinoma cell lines SW480 and SW620 [18]. The extract induced apoptosis in both SW480 and SW620 cells, with cell cycle arrest occurring in the S, G2/M phases, and an increase in the SubG0/G1 population, respectively, without inducing significant mitochondrial membrane damage or oxidative stress. However, in this study, no investigation into specific proteins modulated during the apoptosis process was conducted, and no indication of the type of apoptosis was provided. Additionally, the mechanisms were evaluated using an aqueous extract of Andean berry, which does not reflect the way the fruit could be consumed. Research on the full food matrix has gained relevance due to an understanding of food products not only as compositions of bioactive compounds but also as their relationships and behavior within the food structure, which greatly influences their biological and technofunctional effects [19,20].

Although previous studies have evaluated Andean berry in colon cancer cells, none have investigated the mechanistic pathways underlying its cytotoxic, antiproliferative, and pro-apoptotic effects. Moreover, there are no reports of proteomic modulation of apoptosis induced by Andean berry juice (ABJ), in comparison between early-stage and metastatic human colon cancer cell lines. Additionally, most research on Andean berry has been conducted using purified extracts, without reflecting on how the fruit could be regularly consumed as juice and its biological impact under these conditions. Therefore, this research aimed to evaluate a widely consumed food matrix, specifically berry juice, for its ability to induce cell death in human colon adenocarcinoma SW480 cells and their metastatic derivative, SW620, through proteomic analysis and investigation of the mechanisms underlying the apoptotic process.

## 2. Results

### 2.1. Evaluation of the Cytotoxic Activity of Andean Berry Juice (ABJ) in Cell Lines

**Figure 1:**
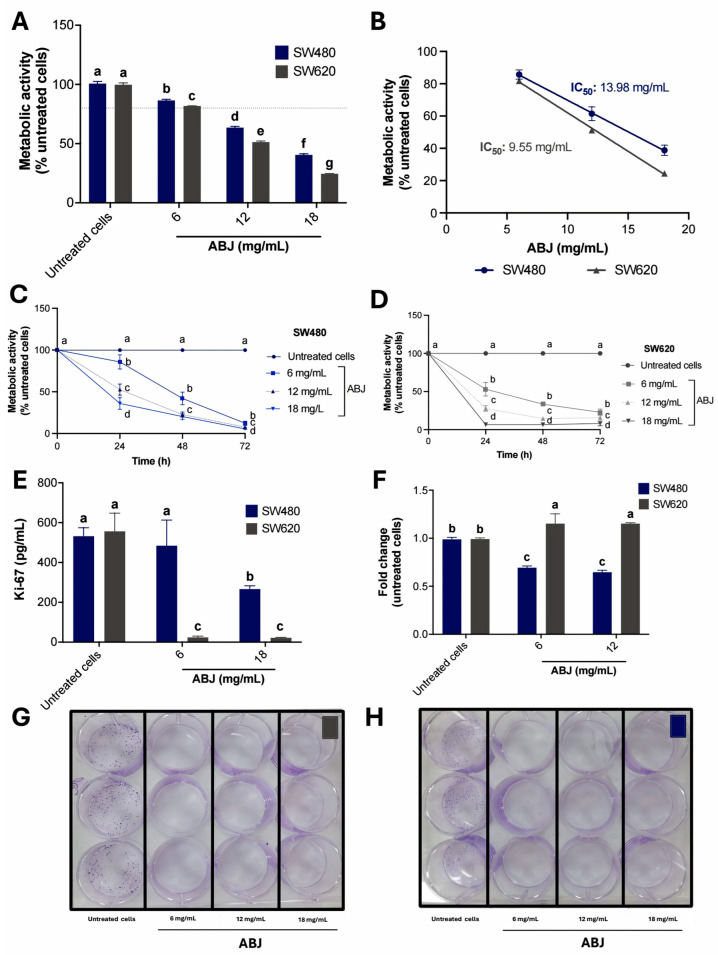
Effect of Andean berry (*Vaccinium meridionale* Swartz) juice (ABJ) on the metabolic activity, proliferation, and morphology of human SW480 and SW620 cells. (**A**) Impact of ABJ on the metabolic activity of SW480 and SW620 cells; (**B**) Half-inhibitory concentration (IC_50_) quantification; (**C**) Antiproliferative effect of ABJ on SW480; (**D**) Antiproliferative impact of ABJ on SW620 cells; (**E**) Quantification of Ki-67 protein; (**F**) Cells’ granularity; (**G**) Clonogenic efficiency of SW480 cells under ABJ treatments; (**H**) Clonogenic efficiency of SW620 cells treated with ABJ concentrations. The results in (**A**,**C**–**F**) were expressed as the mean ± S.D. of at least two independent experiments in triplicate. Different letters express significant differences (*p* < 0.05) by Tukey–Kramer’s test. Assessments in A-D were conducted using the sulforhodamine B (SRB) assay. For the IC_50_ calculation in B, a regression curve adjusted to biological models was used in the dose–response utility of GraphPad Prism v. 10.0. Untreated cells corresponded to either SW480 or SW620 cells in 2% FBS-DMEM. ABJ: Andean berry (*Vaccinium meridionale* Swartz) juice; SW480: Human early-stage colon cancer cells; SW620: Human metastatic colon cancer cells. Treatments in F were assayed at 6 and 12 mg/mL ABJ, since cells at 18 mg/mL exhibited severe damage.

Figure 1 shows the effects of Andean berry (*Vaccinium meridionale* Swartz) juice (ABJ) on the metabolic activity (Figure 1A,B), proliferation (Figure 1C,D), the Ki-67 protein levels (Figure 1E), granularity (Figure 1F), and the clonogenic efficiency (Figure 1G,H) of SW480 and SW620 cells. 

Significant cytotoxic effects were observed at ABJ concentrations above 12 mg/mL, with greater cytotoxicity detected in SW620 cells, as reflected by an IC_50_ value that was 31.68% lower than observed in SW480 cells (Figure 1B) (*p* < 0.001). The IC_50_ values were 13.98 ± 0.34 mg/mL (95% CI: 13.12 to 14.84 mg/mL) for SW480 cells, and 9.55 ± 0.05 (95% CI: 9.44 to 9.66 mg/mL) for SW620 cells.

As observed during the 72 h treatment period with ABJ (Figure 1C,D), SW620 cells displayed markedly lower metabolic activity over time than SW480 cells. Although all ABJ treatments decreased Ki-67 levels in both cell lines, SW620 cells showed substantially lower values at 6 and 12 mg/mL (−95.06% and −91.81%, respectively) (Figure 1E). In contrast, granularity showed the opposite trend (Figure 1F), with SW620 cells exhibiting greater fold changes than SW480 cells (*p* < 0.05). Similar effects were observed in clonogenic assays performed in 24-well plates, in which no colony formation was detected at any ABJ concentration in either SW480 or SW620 cells (Figure 1G,H). These findings were quantified as absolute clonogenic efficiency (ACE) and relative clonogenic efficiency (RCE) and are presented in Supplementary Table S1.

### 2.2. Impact of ABJ on Apoptosis and Cell Cycle Distribution of SW480 and SW620 Cells

**Figure 2:**
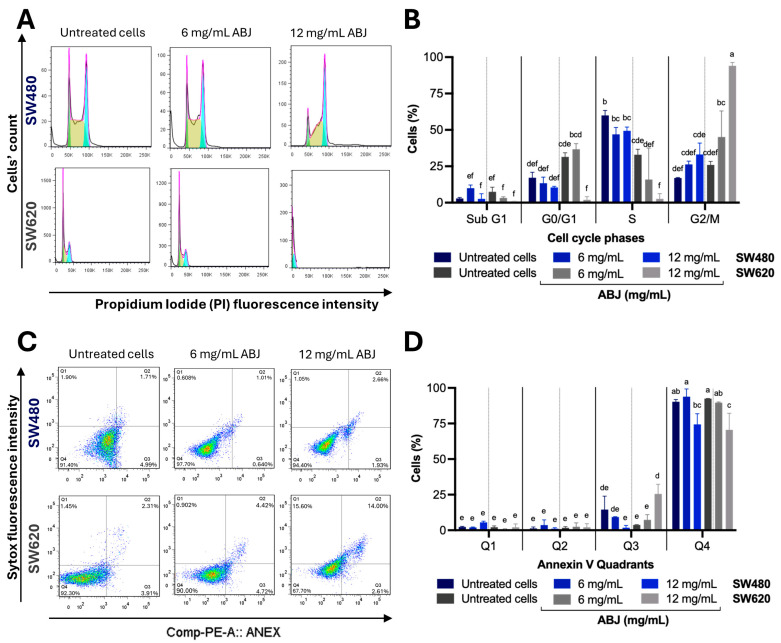
Evaluation of the impact of Andean berry (*Vaccinium meridionale* Swartz) juice (ABJ) on apoptosis and cell cycle distribution of SW480 and SW620 cells. (**A**) Representative pictures of cells’ plot by flow cytometry of the quadrants’ distribution of cells as necrotic/apoptotic/live cells; (**B**) Quantification of the percentage of cells on each quadrant; (**C**) Evaluation of the cell cycle by propidium iodide (PI) staining showing representative pictures of the cell plots; (**D**) Quantification of the number of cells (%) for each stage of the cell cycle. The results were expressed as the mean ± S.D. of at least two independent experiments in triplicate. Different letters indicate significant differences (*p* < 0.05) between all treatments by Tukey–Kramer’s test. Untreated cells corresponded to either SW480 or SW620 cells in 2% FBS-DMEM. For the apoptosis assessment in B, Q1: necrotic cells; Q2: late apoptotic cells; Q3: early apoptotic cells; and Q4: live cells. For the cell cycle analysis in D, Sub G1: Cells with fragmented DNA before the G1 phase; G0/G1: Cell growth and preparation for DNA synthesis; S: Duplication of the cells’ DNA; G2/M: Stage between protein synthesis for division and the mitotic stage. Untreated cells corresponded to either SW480 or SW620 cells without treatment (2% FBS DMEM). ABJ; Andean berry (*Vaccinium meridionale* Swartz) juice; PI: Propidium iodide; SW480: Human early-stage colon cancer cells; SW620: Human metastatic colon cancer cells.

Exposure of phosphatidylserine on the cell membrane is associated with apoptosis and can be detected by Annexin V staining, whereas Sytox Green staining allows the discrimination of apoptotic and necrotic cells. The representative dot plots (Figure 2A) show the displacement of cells toward quadrants Q_2_ (late apoptotic cells) and Q_3_ (early apoptotic cells), as indicated by annexin V incorporation. Quantification of the percentage of cells in each quadrant (Figure 2B) revealed higher cell populations in Q3 and Q4 following ABJ treatment, suggesting that ABJ promotes the transition from live to early apoptotic cells. Similar behavior was observed in both SW480 and SW620 cells, although the highest ABJ concentration induced the strongest pro-apoptotic effect in SW620.

The cell cycle consists of four sequential stages during which cells replicate their DNA and subsequently divide. Quantification of cellular DNA after 24 h of treatment with the ABJ concentrations of 0, 6, and 12 mg/mL promoted the distribution of cells among the G0/G1, S, and G2/M phases. Figure 2C shows representative histograms for the cell lines, and Figure 2D displays the quantification of the number of positive cells at each cell cycle stage. Metastatic SW620 cells predominantly accumulated in the G2/M phase, whereas SW480 cells accumulated in the S-phase, indicating differential modulation of the cell cycle progression between the two cell lines.

### 2.3. Analysis of Mitochondrial Membrane Integrity and Activity in ABJ-Treated Cells

**Figure 3:**
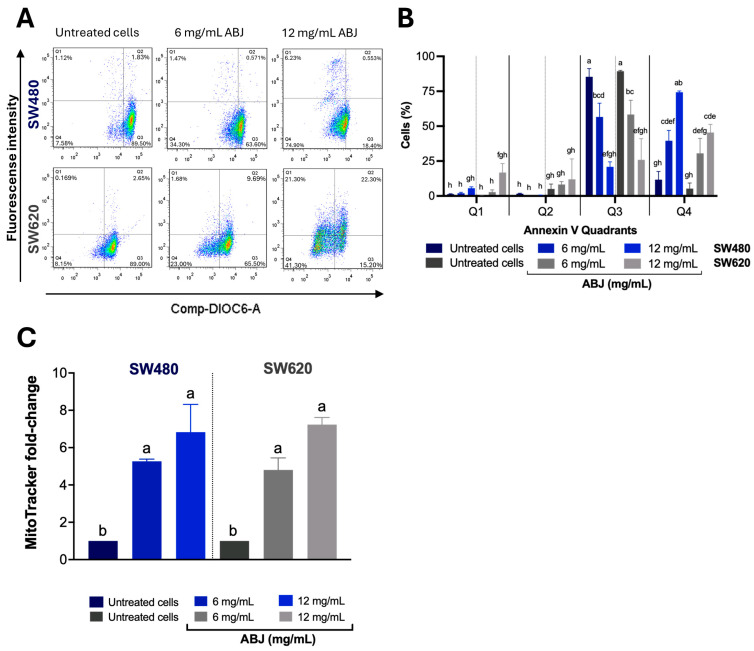
Impact of Andean berry (*Vaccinium meridionale* Swartz) juice (ABJ) on mitochondrial membrane integrity and activity in SW480 and SW620 cells. (**A**) Mitochondrial membrane potential (ΔΨm) representative pictures; (**B**) Quantification of the mitochondrial membrane potential by distribution of the cells on each quadrant; (**C**) MitoTracker fold-change quantification. The results were expressed as the mean ± S.D. of at least two independent experiments in triplicate. Different letters indicate significant differences (*p* < 0.05) between all treatments by Tukey–Kramer’s test. Untreated cells corresponded to either SW480 or SW620 cells in 2% FBS-DMEM. Q1 (DiOC6−/PI+): dying cells (nonapoptotic/necrotic), low ΔΨm, and low membrane integrity; Q2 (DiOC6+/PI−): Late apoptosis, high ΔΨm, and low membrane integrity; Q3 (DiOC6+/PI−): High ΔΨm and proper membrane integrity; Q4 (DiOC6−/PI−): low ΔΨm, and good membrane integrity. ABJ: Andean Berry (*Vaccinium meridionale* Swartz) juice; DiOC6: 3,3′-dihexyloxacarbocyanine iodide; PI: propidium iodide; SW480: Human early-stage colon cancer cells; SW620: Human metastatic colon cancer cells.

A reduction in mitochondrial membrane potential (ΔΨm) is an indicator of early apoptosis in response to treatment-induced damage. Staining with 3,3-dihexylocarbocyanine iodide (DiOC6) allows the mitochondrial response of cells to treatment to be evaluated. Double staining with propidium iodide (PI) allows for the differentiation of live or dead cells with loss of mitochondrial membrane integrity. Figure 3A presents representative images of cells positive for DiOC6-positive cells, where a transition from Q3 to Q4 is observed as the ABJ concentration increases, as confirmed by the quantification of the percentage of cells (Figure 3B), where those quadrants displayed the highest accumulation of cells, with values being higher in SW480 than SW620. The results indicated that ABJ treatments promote a mild mitochondrial membrane depolarization. The results from the positive control (50 mM H_2_O_2_) confirmed the increased accumulation of cells in the Q2 and Q3 quadrants (Supplementary Figure S1). Despite a mild membrane polarization, results from MitoTracker staining of positive cells showed that all ABJ treatments significantly increased the number of stained cells (*p* < 0.05) (Figure 3C), suggesting altered mitochondrial activity.

### 2.4. Human Apoptosis Proteome Analysis in ABJ-Treated Cells

**Figure 4:**
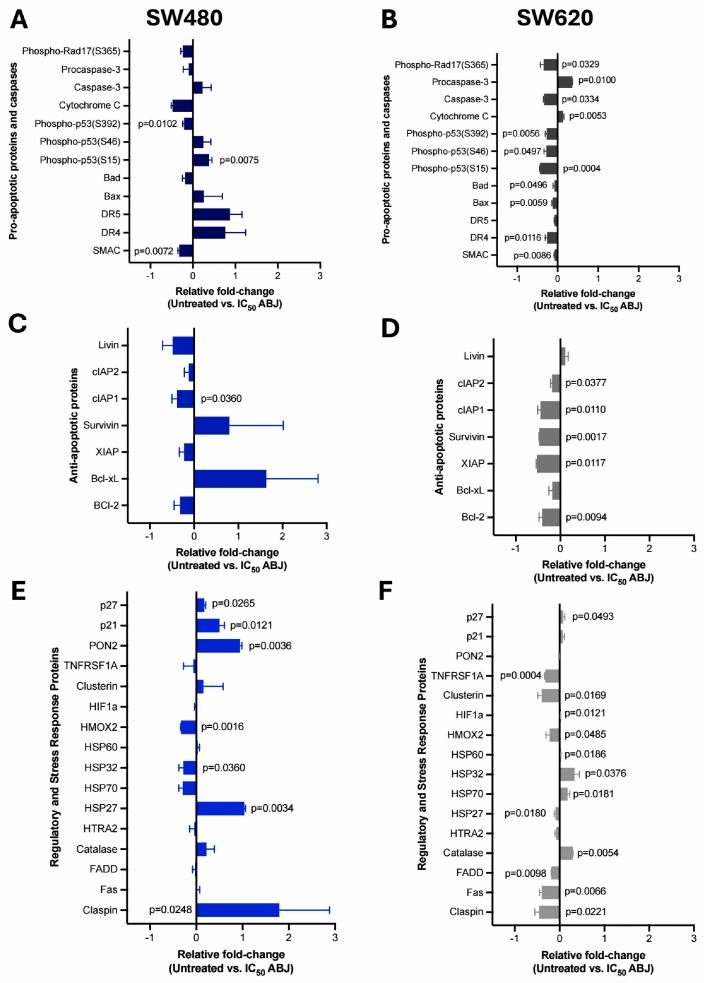
Assessment of Andean berry (*Vaccinium meridionale* Swartz) juice (ABJ) effect on the relative fold-change in assessed cytokines from the apoptotic process in SW480 and SW620 cells. (**A**) Pro-apoptotic proteins and caspases modulated by IC_50_ ABJ in SW480 cells; (**B**) Pro-apoptotic proteins and caspases modulated by IC_50_ ABJ in SW620 cells; (**C**) Anti-apoptotic proteins modulated by IC_50_ ABJ in SW480 cells; (**D**) Anti-apoptotic proteins modulated by IC_50_ ABJ in SW680 cells; (**E**) Regulatory and stress-response proteins modulated by IC_50_ ABJ in SW480 cells; (**F**) Regulatory and stress-response proteins modulated by IC_50_ ABJ in SW620 cells. The results were expressed as the mean ± S.D. of at least two independent experiments in triplicate. Only significant *p*-values were indicated in the figures, assessed by Student’s *t*-test. The significance was assessed by comparing the cytokine’s relative intensity between the untreated (2% FBS DMEM-only) cells and those treated with the half-inhibitory concentration of Andean berry (*Vaccinium meridionale* Swartz) juice (IC_50_ ABJ). Bad: Bcl-2-associated death promoter; Bax: Bcl-2 associated X protein; BCl-2: B-cell lymphoma 2; Bcl-xL: B-cell lymphoma-extra-large; cIAP1: Cellular inhibitor of apoptosis protein 1; cIAP2: Cellular inhibitor or apoptosis protein 2; DR4: Death receptor 4; DR5: Death receptor 5; FADD: Fas-associated death domain protein; Fas (CD95): FS-7 associated protein; HIF1a: Hypoxia-inducible factor 1, alpha subunit; HMOX2: Heme oxygenase 2; HSP27: Heat-shock protein 27; HSP32: heat-shock protein 32; HSP60: Heat-shock protein 60; HSP70: Heat-shock protein 70; HTRA: High-temperature requirement A; PON2: Paraoxonase 2; SMAC: Second mitochondria-derived activator of caspase; SW480: Human early-stage colon cancer cells; SW620: Human metastatic colon cancer cells; TNFRSF1A: Tumor necrosis factor receptor superfamily, member 1A; XIAP: X-linked inhibitor of apoptosis protein.

The expression of apoptosis-related proteins (Figure 4) was evaluated in SW480 and SW620 cells treated with the IC_50_ concentration of ABJ for each cell line, as determined by the results shown in Figure 1B. Relative fold changes in protein expression were calculated relative to untreated cells. To facilitate visualization and interpretation of the analyzed proteins, they were grouped into three categories: pro-apoptotic proteins and caspases (Figure 4A,B), anti-apoptotic proteins (Figure 4C,D), and proteins related to regulation and stress response (Figure 4E,F).

Overall, SW620 cells showed a greater number of proteins with significant differences in expression (*p* < 0.05) between untreated and ABJ-treated cells; however, the observed fold changes were generally lower than those detected in SW480 proteins. Among the pro-apoptotic proteins and caspases (Figure 4A,B), phospho-p53 (S392) and SMAC showed similar expression patterns in both cell lines. In contrast, procaspase-3, cytochrome C, Bcl-2-associated death promoter (Bad), Bcl-2-associated X protein (Bax), death receptor 4 (DR4), and death receptor 5 (DR5) showed opposite trends, suggesting different pro-apoptotic mechanisms in the two cell lines.

Within the anti-apoptotic protein cluster (Figure 4C,D), SW620 showed decreased expression of several anti-apoptotic proteins, such as Survivin, X-linked inhibitor of apoptosis (XIAP), and B-cell lymphoma extra-large protein (Bcl-xL), indicating differential responses to IC_50_ ABJ treatment between the two cell lines. Regarding proteins associated with regulation and stress response (Figure 4E,F), ABJ treatment increased the amount of p27, p21, Paraoxonase-2 (PON2), Clusterin, heat-shock protein 27 (HSP27), and Claspin in SW480 cells, whereas the opposite trend was observed in SW620 cells.

Comparisons of the relative fold-change in each protein with their respective untreated counterparts between SW480 and SW620 cells revealed significant differences (*p* < 0.05) for 19 proteins (Supplementary Table S2). Among these proteins, only BcL-xL was considered upregulated (fold-change ≥ 1.50), whereas all the other proteins were downregulated (fold-change ≤ 0.67).

**Figure 5:**
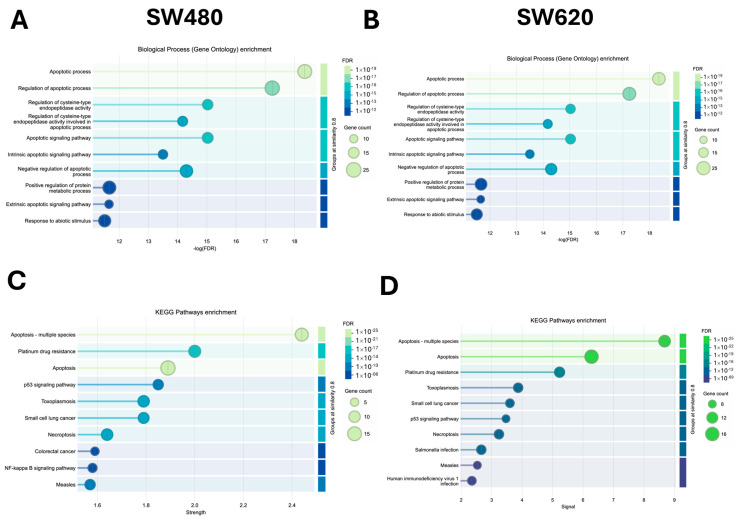
Bioinformatic analysis of the apoptosis-related proteins modulated by ABJ in SW480 and SW620 cells. (**A**) Biological process (gene ontology) enrichment in SW480 cells; (**B**) Biological process (gene ontology, GO) enrichment in SW620 cells; (**C**) Kyoto Encyclopedia of Genes and Genomes (KEGG) enrichment in SW480 cells; (**D**) KEGG Enrichment in SW620 cells. SW480: Human early-stage colon cancer cells; SW620: Human metastatic colon cancer cells.

A bioinformatic analysis of the evaluated proteins was conducted using the STRING platform (Figure 5). The main enriched biological processes identified (Figure 5A,B) included the apoptotic process and the regulation of the apoptotic process, and both extrinsic and intrinsic apoptotic mechanisms were highlighted in SW620 cells. Hub proteins were identified according to node degree centrality within the STRING network, where caspase 3 and cytochrome 3 were shown as the most interconnected nodes (node degree: 23), followed by Bcl-2 (degree: 22) and the Bcl-2-like protein 1 (BCL2L1) (Supplementary Table S3). Additionally, the generated protein network for each cell line is presented in Supplementary Figure S2.

Regarding Kyoto Encyclopedia of Genes and Genomes (KEGG) pathway enrichment, apoptosis, the p53 signaling pathway, and necroptosis are among the highest-ranked pathways based on false discovery rate (FDR) and the program’s calculated strength (Figure 5C,D). Only pathways with FDR < 0.05 were considered as significantly enriched (Supplementary Tables S4 and S5).

### 2.5. Principal Component Analysis (PCA) of the Observed Biological Processes Modulated by ABJ

**Figure 6:**
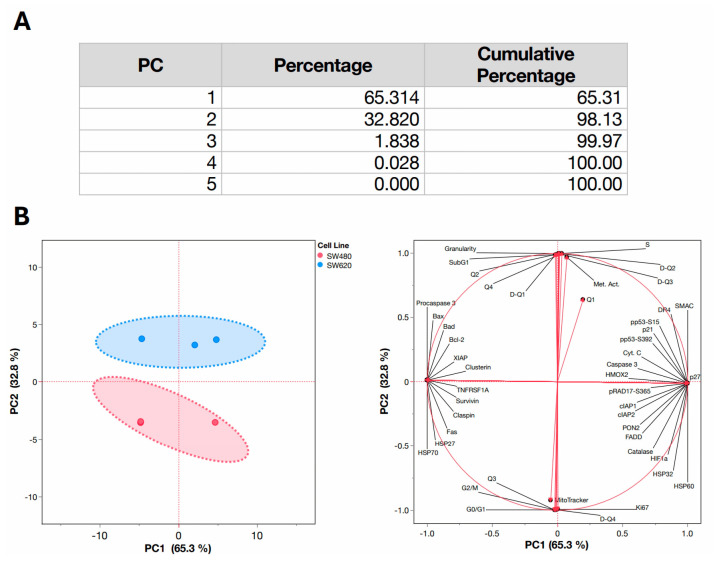
Principal component analysis (PCA) for the assessed variables impacted by Andean berry (*Vaccinium meridionale* Swartz) juice on SW480 and SW620 cells. (**A**) Individual and cumulative percentages covered by each principal component (PC); (**B**) Scatter plot and loading plots for the first and the second component after the evaluation of all variables.

Results from the PCA analysis (Figure 6) showed that only 2 components explained > 90% of the total variation (Figure 6A). Two differentiated clusters were observed for SW480 and SW620 cells (Figure 6B), where the ABJ treatment on SW480 cells mostly impacted the distribution of cells in apoptosis (Q2–Q4), three quadrants of the DiOC6 staining (DQ1–DQ3), granularity, metabolic activity, and two stages of the cell cycle (SubG1 and S). On the other hand, ABJ treatment primarily modulated the Q3 quadrant of apoptosis, the G0/G1 and G2/M stages of the cell cycle, and the D-Q4 stage, as indicated by DiOC6, Ki-67, and MitoTracker staining in SW620 cells. Principal component 1 (PC1) variation was primarily driven by Ki-67, Q2 of the apoptosis process, and D-Q2 from DiOC6 staining in the positive direction, whereas tumor necrosis factor receptor superfamily member 1A (TNFRSF1A), Clusterin, and heat-shock protein 70 were in the negative direction. For PC2, Bax, Bad, and procaspase proteins explained their positive variation, and the Q3 quadrant of apoptosis, G2/M and G0/G1 stages of the cell cycle, and the D-Q4 quadrant from DiOC6 staining explained their negative variation (Supplementary Table S6).

## 3. Discussion

This study demonstrated differential programmed death induction (apoptosis) in an in vitro model of colorectal adenocarcinoma (CRC), using the SW480 cell line and its metastatic derivative, SW620, treated with Andean berry juice (ABJ) (*Vaccinium meridionale* Swartz). The results showed that ABJ significantly decreased cell viability after 24 h of exposure to different juice concentrations. The IC_50_ values reported in this study are close to those reported in other studies that evaluated different concentrations of ABJ in SW480 and SW620 cells [21]. In other studies evaluating cytotoxicity using the same cell lines, the metastatic cells showed greater sensitivity to the juice, with lower IC_50_ values than those reported for SW480 in this investigation [13]. Other products, such as vinegar, nectar, and even green and black tea obtained from the leaves of this plant species, have been shown to reduce cellular metabolic activity and promote antiproliferative activity in these cell lines [22]. Similar results have also been obtained in the combination of ABJ with Aspirin, a well-known anti-inflammatory drug [21,23]. The cytotoxic, antiproliferative, and antitumor potential of *V meridionale* has also been evaluated in in vitro models of various cancers, including fibrosarcoma (HT1080) cells, transformed leukemia cells (MOLT4 cell line), and HT29 colorectal adenocarcinoma cells [16,24,25]. Additionally, the antitumor effect of ABJ has been demonstrated by a drastic, significant decrease in cell cloning capacity reported in other studies using SW480 cells [23]. The results of this study show the potential effects of this fruit against various cancers in vitro. It is important to note that this research was intended to assess undigested ABJ juice, based on previous reports from our research group testing several ABJ concentrations in either SW480 or SW620 cells. However, from a physiological perspective, food products are subjected to gastrointestinal conditions that can enhance their biological effects by the activity of digestive enzymes, which can release or biotransform phytochemicals from the food matrix [26].

The Ki-67 protein is a marker of cell proliferation, and an increase in its concentration is associated with greater proliferative capacity [27]. Together with p53 expression, it is related to early relapse and distant metastasis in colorectal cancer patients [28]. The results from the amount of protein found in cells after ABJ treatment indicated that SW620 cells are more prone to accumulate Ki-67, consistent with previous reports of its primary association with metastatic cells and excessive cell proliferation in colorectal cancer [29], yet ABJ showed limited ability to reduce proliferation in this cell line. Moreover, ABJ failed to reduce cell size or granularity in SW620 cells. However, these cells are readily misclassified as granulocytes due to their size and high variability in optical path delay when examined by flow cytometry [30].

When evaluating an agent with potential to reduce cancer risk, it is important to assess its capacity to modulate the cell cycle, as assessing both apoptosis and the cell cycle provides information about cancer molecular pathogenesis and how tumor cells respond to therapy. In SW480 cells, the percentage of cells in S phase increased, then decreased in G2/M phase as ABJ concentration increased. These results are consistent with those reported in a previous study evaluating ABJ, in which an increase in the percentage of SW480 cells in the S phase was observed at an ABJ concentration of 18 mg/mL. It was proposed that ABJ can modulate mitotic division [18]. On the other hand, in SW620 cells, the percentage of cells in the G2/M phase increased, indicating that ABJ modulates the continuity of the cycle towards this phase. It has been proposed that an elevated activity of G2/M-related genes is an indicator of increased aggressivity and metastatic potential [31], although this has been proposed for estrogen receptor-positive breast cancer cells [32].

In the present work, the ability of ABJ to induce apoptosis in the two cell lines was evaluated to compare the responses of cells that share a monoclonal origin but exhibit significant phenotypic differences, as SW620 cells were originally derived from a lymph node metastasis of the same tumor that gave origin to SW480 cells [33]. The Annexin V and Sytox Green assays showed that ABJ induces phosphatidylserine exposure without membrane depolarization. These results are consistent with those reported in other studies [18,21], in which the evaluated concentrations of an aqueous extract of the Andean berry induced apoptosis without mitochondrial damage in the two cell lines, but increased reactive oxygen species [34]. A comprehensive evaluation of the effect of procyanidins from different berries, such as wild lowbush berry (*Vaccinium myrtillus*), highbush blueberry (*Vaccinium corymbosum*), lingonberry (*Vaccinium vitis-idaea*), raspberry (*Rubus idaeus*), wild blackberry (*Rubus fruticosus*), thornfree blackberry (*Rubus fruticosus* ‘Thornfree’), redcurrant (*Ribes rubrum*), gooseberry (*Ribes uva-crispa*), blackcurrant (*Ribes nigrum*), jostaberry (*Ribes nidigrolaria*), and cranberry (*Vaccinium macrocarpon*) showed apoptosis induction in SW480 and SW620 cells in a more potent manner than apple procyanidins. Still, these compounds did not sensitize either cell line to TRAIL-induced apoptosis [35]. Ellagic acid, one of the phenolic compounds identified in ABJ, has been linked to chemosensitivity effects on human colorectal carcinoma cells. Its evaluation in HT-29 and SW480 in a 2.5–25 µg/mL (which is much lower than the reported values for ABJ) decreased the proliferation of these cells, enhanced the Bax: Bcl-2 ratio, triggered caspase-3 activation, and promoted apoptosis in these cells, making them more susceptible to the treatment with well-known chemotherapy agents like 5-fluorouracil [36]. Gallic acid, also present in ABJ, prevents cellular proliferation and arrests the cell cycle in G0/G1 by decreasing cyclin D1 level, results that have been observed in human Caco-2 and HCT-15 cells, also cells from primary tumor, low metastatic potential, and exhibiting mutations in the Kirsten rat sarcoma viral oncogene homolog (KRAS) protein like SW480 cells [37,38]. More recently, chlorogenic acid concentrations up to 2000 µM decreased the G1 phase and increased the subG1 phase in SW480 cells, suggesting induction of apoptosis, as fragmented DNA was detected by the authors [39].

An additional ability of different ABJ concentrations to induce cell death was evaluated by staining for outer and mitochondrial membranes, assessing expression of markers associated with apoptotic signaling pathways, and visualizing apoptotic cell nuclei. MitoTracker dyes have been found to be released from mitochondria, like other dependent dyes, and this release could be affected by the intracellular ROS levels, cellular redox state, membrane potential, or mitochondrial stress [40]. Hence, the observed MitoTracker values after ABJ treatments do not depend solely on mitochondrial mass but also on additional factors that could be confirmed by DiOC6 analyses, which were independent assays evaluating the dependence of mitochondrial membrane potential on mitochondrial alterations [41,42]. Previously, no mitochondrial damage was reported in ABJ-treated SW6480 and SW620 cells at ABJ concentrations higher than those used in this research, and ABJ did not induce ROS production in the cells [18]. However, additional mitochondrial mechanisms were observed following MitoTracker analysis, with marked mitochondrial damage evident after ABJ treatments. In SW480 and HT-29 cells, chlorogenic acid has induced marked mitochondrial ROS production, up to 3-fold, at concentrations ranging from 0 to 2000 µM, thereby increasing activation of proapoptotic molecules, such as caspase-3 [39].

The evaluation of proteomic profiles of SW480 and SW620 cells treated with ABJ complemented the results from the Annexin V assay and protein modulation. Based on the observed results, ABJ predominantly activated the extrinsic apoptotic pathway in SW480 cells, while the intrinsic apoptotic pathway dominated in SW620 cells. As observed, there was an overexpression of proapoptotic markers of the extrinsic pathway, such as Bax, phospho-p53 (S15), phospho-p53 (S46), caspase 3, TRAIL/DR4, and DR5 in IC_50_-treated SW480 cells, but only phospho-p53 (S15) was significantly higher than in untreated SW480 cells. In addition, there was activation of cell cycle modulating proteins, such as claspin (fold-change > 1.50), and decreased p21 protein (fold-change < 0.67), as well as response markers against cellular stress, such as PON2 and HSP27. The activity of all these markers confirms the results obtained in the Annexin V, DiOC6, and MitoTracker assays.

Previous research from our group [23] showed overexpression of TRAIL-DR4, TRAIL-DR5, PON2, and CDKN1A proteins in SW480 cells treated with 30% *v*/*v* ABJ (36 mg/mL) and Aspirin, underexpression of catalase, and an increase in caspase 3 expression at 12 mg/mL ABJ. Despite being metabolically distinct cell lines, another report found no differences in catalase activity in human HT29 colorectal cancer cells treated with 24.69% of the fermented non-digestible fraction of ABJ (equivalent to 1.22 mg/mL) [16]. Although in this research an independent assessment of TRAIL DR4 showed no differences between the untreated and the treated cells (6 and 12 mg/mL ABJ), non-significant increases (*p* > 0.05) were presented for SW480 and significant decreases (*p*: 0.0116) were shown in SW620 (Figure 4), which is similar to our observed trends (*p* > 0.05) (Supplementary Figure S3). Moreover, the modulation of proteins in SW480 is partially consistent with the observed cell-cycle arrest in S-phase, since p27 is a cyclin-dependent kinase inhibitor found in early-stage colorectal cancer patients, which promotes cell-cycle arrest, particularly at G1/S, by inhibiting CDK2-cyclin E and CDK4-cyclin D complexes [43].

Regarding SW620 cells, the expression of these apoptosis-determining markers was observed through the overexpression of the non-active form of caspase 3 (extrinsic apoptosis) and the release of cytochrome c (intrinsic apoptosis), in addition to the underexpression of anti-apoptotic markers such as Bcl-2 and XIAP, confirming a dominance of the intrinsic mitochondrial proapoptotic pathway. In cell cycle regulation of SW620 cells by ABJ treatment, decreased phosphoRad17 and Claspin are indicative of dysregulation or mitotic stress rather than a properly activated checkpoint that arrests the cell cycle in G2/M, since Ras17 phosphorylation in Ser635 and Ser645 is essential for its function and promotes claspin phosphorylation, needed to mediate G2 arrest in response to DNA damage [44]. In addition, decreased Survivin levels could support the idea of impaired mitotic regulation, since reduced Survivin levels suggest a disruption of its required association with the microtubules of the mitotic spindle, thereby impairing mitotic regulation [45].

The relationship to specific molecular pathways linked to apoptosis was confirmed through bioinformatics enrichment analysis, which also supported most of these conclusions, drawn from studies with berries and molecular markers of apoptosis. The results from the nodes analysis identified proteins with a central role in mitochondrial apoptosis and caspase activation in response to ABJ. The differential expression of these proteins allows a differential molecular response to be observed between the two cell lines treated with ABJ at 6 and 12 mg/mL. This needs to be investigated at a deeper level by inhibiting specific markers, allowing the ability of ABJ to induce apoptosis through intrinsic or extrinsic pathways to be distinguished.

In silico analysis prediction of the impact of specific compounds on protein markers of apoptosis provides insights into molecular interactions between phytochemicals. Predicting the molecular interaction between apoptosis markers and the phytochemicals present in the Andean berry, such as chlorogenic acid, caffeic acid, and cyanidin 3 glucoside [46,47] (Supplementary Table S7) could also explain the effect of ABJ on the activation or inhibition of the proteins of interest to be characterized. Previous findings have shown that some of the ABJ compounds evaluated exhibit affinity for proteins involved in apoptosis, including Bcl-2, cytochrome c, and caspase-3. Particularly, cyanidin-3-glucoside, which is present in high concentrations in the berry, exhibits elevated coupling affinity for receptors such as TRAIL-DR4, which, when activated, triggers caspase activity that executes apoptosis [48]. Moreover, individual evaluation of selected phenolic compounds, such as gallic acid and chlorogenic acids, has shown anti-inflammatory effects by reducing nitrite production and intracellular reactive oxygen species in lipopolysaccharide-stimulated RAW 264.7 macrophages [49], suggesting potential synergistic effects between pro-inflammatory and cancer markers, which merit further research using biologically relevant concentrations. Since it is known that ABJ digestion allows the absorption of bioaccessible compounds, such as gallic acid, chlorogenic acid, caffeic acid, ellagic acid, and rutin, together with other compound classes like oligosaccharides (raffinose, stachyose, and verbascose) [17], the joint activity of these compounds could definitely be contributing to the observed effects in colorectal cancer lines from this research.

The PCA analysis aimed to integrate the results and provide greater discrimination between SW480 and SW620 cells, which were also applied to selected cell lines, linking to the composition of the molecular entities used to challenge the cells. The dimensional reduction in components linked to SW480 cells is more closely associated with cell cycle progression, metabolic activity, and early cell cycle stages. In contrast, SW620 cells were associated with intermediate stages of the cell cycle, as indicated by Ki-67 and MitoTracker.

## 4. Materials and Methods

### 4.1. Preparation of Juice from Freeze-Dried Andean Berry (Vaccinium meridionale Swartz) Juice (ABJ)

A very well-standardized Andean berry juice (ABJ) was prepared following previously reported methodologies [17]. This involved carrying out chemical characterization of the juice, in which the total phenolic compounds (3570 ± 260 mg gallic acid equivalents/100 mL), total flavonoids (2310 ± 20 mg of (+)-catechin equivalents/100 mL), and total monomeric anthocyanins (129 ± 20 mg cyanidin-3-O-glucoside/100 mL). The individual phenolic compounds, identified by high-performance liquid chromatography coupled to diode-array detection (HPLC-DAD), included gallic acid (658.45 ± 8.80 μg/g), chlorogenic acid (35.31 ± 2.90 μg/g), caffeic acid (11.52 ± 0.10 μg/g), ellagic acid (69.31 ± 5.00 μg/g), *p*-coumaric acid (6.50 ± 0.20 μg/g); 3,4-dihydroxybenzoic acid (809.23 ± 0.87 μg/g); 2-hydroxycinnamic acid (0.38 ± 0.01 μg/g), (+)-catechin (41.32 ± 0.10 μg/g), rutin (111.23 ± 0.30 μg/g), morin (133.88 ± 7.70 μg/g), and kaempferol (14.57 ± 0.10 μg/g) [23]. Novel analysis of some of these compounds found in the juice is presented in Supplementary Table S8 and Supplementary Figure S4.

Furthermore, the juice exhibited antioxidant capacity in vitro, as demonstrated by ferric reducing antioxidant power (FRAP) and 2,2-diphenyl-1-picrylhydrazyl (DPPH) inhibition values of 127.60 and 35.43 µmol Trolox equivalents/mL, respectively [18].

### 4.2. Cell Culture

Human SW-480 colorectal adenocarcinoma cells [SW-480] (ATCC CCL-228) and their metastatic derivatives SW-620 [SW-620] (ATCC CCL-227) were obtained from American Type Culture Collection (ATCC, Manassas, VA, USA). The cells were maintained in Dulbecco’s Modified Eagle’s Medium (DMEM) high-glucose (25 mM), supplemented with L-glutamine, fetal bovine serum (FBS, 10% *v*/*v*), and antibiotics (100X, 1% *v*/*v*). Unless indicated otherwise, all reagents used for the cell culture maintenance were acquired from Gibco (Thermo Fisher Scientific, Waltham, MA, USA). Cells were maintained in a humidified 5% CO_2_ atmosphere at 37 °C.

#### 4.2.1. Assessment of the Metabolic Activity of the Cells Exposed to Andean Berry (*Vaccinium meridionale* Swartz) Juice (ABJ)

From both lines, 2 × 10^4^ cells/well were cultured in 96-well plates. After 24 h, different concentrations of ABJ (0, 6, 12, and 18 mg/mL) were added. The selection of these concentrations was based on a previous study in which in vitro gastrointestinal digestion of ABJ was performed. In that study, the effective IC_50_ of the digested extract was 19.30% *v*/*v*, whereas that of the nondigested whole juice was 19.8% *v*/*v*, corresponding approximately to 24 mg/mL for the undigested juice [17]. Furthermore, additional research conducted by our group demonstrated that ABJ concentrations near 19.92% (equivalent to 23.9 mg/mL) induced cytotoxicity in SW480 cells [23]. After the exposure time had elapsed, the cultures were interrupted by adding 50 μL of 50% trichloroacetic acid. They were incubated at 4 °C for 1 h, then the acid was replaced with sulforhodamine B (SRB) (0.4% *w*/*v*, diluted in 1% *v/v* acetic acid) (C_27_H_29_N_2_NaO_7_S_2_ powder, BioReagent, Sigma-Aldrich, St. Louis, MO, USA) for 30 min. Subsequently, the SRB was stirred together with 1% *v/v* acetic acid. For measurement, SRB was solubilized in 200 μL of Tris-HCl buffer (10 mM, pH 10.5) for 20 min. Optical density was measured at 490 nm using a Varioskan plate reader (Thermo Fisher Scientific, Waltham, MA, USA). Five replicates per treatment were performed, and the mean inhibitory concentration (IC_50_) was determined using a statistical regression model. The impact on the metabolic activity was assessed as follows: metabolic activity (%): [(Absorbance sample/Absorbance of negative control] × 100%, where the negative control corresponded to untreated cells (cells cultured in DMEM with 2% FBS). The IC_50_ calculations were performed in GraphPad Prism v. 10 using a non-linear regression (curve-fit) with the 4-parameter logistic (4PL) model for Log (inhibitor) vs. response. The results were expressed as the mean IC_50_ ± S.D. from three independent experiments, and 95% confidence intervals (95% CI) were also calculated.

#### 4.2.2. Impact of Andean Berry (*Vaccinium meridionale* Swartz) Juice on Cell Proliferation

The sulforhodamine B (SRB) assay was used. Both lines were cultured at 3 × 10^3^ cells/well. After 24 h, they were incubated with ABJ (0, 6, 12, and 18 mg/mL) for 0, 24, 48, and 72 h. For the 72 h culture, the medium was reconstituted every 48 h with the respective concentrations. For staining, the process described above was carried out. Five replicates of each treatment were performed, and curves of the cells’ growth over time were plotted. Untreated cells (2% FBS-DMEM) were used as a control.

#### 4.2.3. Evaluation of the Expression of the Ki-67 Protein

For this assay, 1.5 × 10^6^ cells/mL from both cell lines were seeded and incubated at 37 °C and 5% CO_2_. After 24 h, the medium was replaced by the 6 and 18 mg/mL juice treatments. Subsequently, the cells were incubated under the same conditions for 24 h, the supernatant was removed, and the cells were treated according to the manufacturer’s instructions (Human Ki67/MKI67 DuoSet ELISA, R&D Systems Inc., Minneapolis, MN, USA). The optical density at 450 nm was then measured in the Varioskan plate reader (Thermo Fisher, Waltham, MA, USA). The Ki-67 concentration was calculated using the manufacturer-standardized curve.

#### 4.2.4. Assessment of *V. meridionale* Swartz Juice on the Cells’ Granularity

Cell cultures of both lines were treated with ABJ (0, 6, 12, and 18 mg/mL) for 24 h and subsequently observed under a microscope at 40× to assess changes in confluency and cell size. Additionally, the average fluorescence intensity of the parameters was recorded by flow cytometry: SSC (side scatter) for cell granularity. The results were expressed as fold-change relative to the control (untreated cells, growth in 2% FBS-DMEM).

#### 4.2.5. Evaluation of the Effect of Andean Berry (*Vaccinium meridionale* Swartz) Juice (ABJ) on Clonogenic Efficiency

The cells (2.50 × 10^2^) were seeded in 1 mL of maintenance medium (10% FBS DMEM) and incubated for 24 h. The cells were then treated with ABJ (0, 6, 12, and 18 mg/mL) with 3 replicates per treatment and incubated for 24 h. Subsequently, the treatments were replaced with maintenance medium and incubated for 7 days, after which the medium was changed. The adherent cells were fixed with a Carnoy solution (methanol and acetic acid in a 3:1 ratio) and stained with crystal violet (0.5% *w*/*v*) for 1 h. Following this, the dye was removed and washed with distilled water, the plates were left for 24 h, and colony counting was performed under the microscope with the inclusion criterion (1 colony, 30 cells or more). The absolute clonogenic efficiency (ACE) was calculated as the percentage of colonies relative to the number of cells seeded, while the relative clonogenic efficiency (RCE) was calculated as the percentage of ACE_treatments_ relative to ACE_control_.

#### 4.2.6. Analysis of Apoptosis by Sytox/Annexin V Staining

SW480 and SW620 cells were seeded at 1 × 10^6^ cells/mL and incubated for 24 h. After the period, the medium was replaced with the ABJ treatments (0, 6, and 12 mg/mL), and the cultures were incubated for 24 h. After trypsinization, the precipitate was stained with 1 µg SYTOX™ Green (S7020, Invitrogen, Thermo Scientific, Waltham, MA, USA) and Annexin-V-FITC 1X (Annexin V-FITC Apoptosis Detection Kit, APOAF-20TST, Sigma-Aldrich, St. Louis, MO, USA). Fluorescence intensity was measured by flow cytometry, with 10,000 events recorded.

#### 4.2.7. Effect of the Juice on the Cell Cycle

Cell cycle analysis was performed by staining cellular DNA with propidium iodide (PI). Both cell lines were seeded at a concentration of 1 × 10^6^ cells/mL in maintenance medium and incubated for 24 h at 37 °C and 5% CO_2_. After the adherence period, they were treated with ABJ (0, 6, and 12 mg/mL) and incubated for 24 h. Subsequently, they were treated with 500 µL of 1× trypsin-EDTA (Sigma-Aldrich). The suspension was centrifuged (1500× *g*, 10 min), and the precipitate was fixed with 2 mL of cold 70% *v*/*v* ethanol and preserved at 4 °C for 24 h. Finally, the ethanol was removed, and after the last wash, the precipitate was reconstituted in 500 µL of 1× PBS. A mixture of 1 µL PI and RNAase (2 mg/mL) was added, followed by incubation at room temperature for 30 min. The suspensions obtained were analyzed using the FACSCantoII Flow Cytometer (BD Biosciences, Franklin Lakes, NJ, USA), analyzing 10,000 cell events/min.

#### 4.2.8. Analysis of Mitochondrial Membrane Permeabilization by Staining with 3,3-Dihexylocarbocyanine Iodide (DiOC6) and Propidium Iodide (PI)

A cell concentration of 1 × 10^6^ cells/mL of the two cell lines was treated with concentrations of ABJ (0, 6, and 12 mg/mL) for 24 h. Subsequently, all cells were collected by PBS and trypsin washes, stained with 500 nM DiOC6 and PI (2.5 µg/mL), and incubated at room temperature (25 ± 1 °C) for 15 min. Cellular events (10,000/min) were recorded to quantify the number of cells positive for DIOC6/PI staining. A positive control (H_2_O_2_, 50 mM) was included and presented in Supplementary Figure S1.

#### 4.2.9. Analysis of Apoptosis by Staining with MitoTrackerTM Red CMXRos

SW480 and SW620 cells were seeded at a concentration of 1 × 10^6^ cells/mL and treated with ABJ concentrations (0, 6, and 12 mg/mL) for 24 h. Then, the cells were collected after washing with PBS, trypsinized, and centrifuged at 1500× *g* for 4 min. The precipitate obtained was supplemented with MitoTrackerTM Red CMXRos (Thermo Fisher) (150 nM) and incubated at 37 °C for 30 min. After this, the coloring was removed by 3 washes with PBS. The precipitate was resuspended in 250 µL of PBS, and the fluorescence was measured by flow cytometry at 10,000 cell events/min.

#### 4.2.10. Analysis of the Modulation of Proteins

SW480 and SW620 cells were seeded at a concentration of 1 × 10^6^ cells/well in culture dishes and incubated for 24 h at 37 °C. Subsequently, the cells were treated with 12 mg/mL of the juice for 24 h. After the incubation period, the cells were lysed using Halt Protease Inhibitor Cocktail (Thermo Fisher Scientific) (190 µL, dissolved in 9.81mL of the protein inhibitor) in lysis buffer for 30 min at 4 °C. Then, protein concentration was quantified using the Bicinchoninic acid (BCA) kit (PierceTM Thermo Fisher). For each membrane of the Proteome Profiler Human Apoptosis Array kit (R&D Systems, Minneapolis, MN, USA), the same amount of total protein lysate (400 µg) was added, following the manufacturer’s instructions. The images were then quantified using the ChemiDoc XRS+ kit (Bio-Rad, Hercules, CA, USA), and the expression intensity was determined using the Decodon Delta 2D software v. 4.6 (Decodon, Greifswald, Germany). The results were expressed as fold change (FC) relative to the intensity of each protein in its respective control. Fold-change values ≥ 1.5 and ≤0.67 were considered upregulation and downregulation, according to a previous report for the assessment of protein expression in human colorectal cancer cells [50,51]. Although the validation of the protein array was not performed using additional methodologies, reports have confirmed the validity of these assays compared to Western blot for other sets of proteins in SW620 cells [52]. More recently, for a different matrix (lead nanoparticles) and a different human cancer cell line (Caco-2), caspase 3 increases were confirmed using both a protein array and a fluorometric assay kit [53].

For the bioinformatics assessment, evaluations of associated biological processes and Kyoto Encyclopedia of Genes and Genomes (KEGG) pathway enrichment were performed using the STRING (https://string-db.org/, accessed on 1 December 2025) protein–protein interaction networks and functional enrichment analysis, based on the obtained fold-changes in proteins related to the apoptotic process [54].

### 4.3. Statistical Analysis

Unless indicated, the results were expressed, when appropriate, as the mean ± S.D. of at least two independent experiments in triplicate. Then, we carried out one-way analysis of variance (ANOVA) and multiple comparisons using the Tukey–Kramer test after assessing data normality with the Shapiro–Wilk test and homoscedasticity with Bartlett’s test. For all results, a statistically significant difference was considered if *p* < 0.05. Moreover, a principal component analysis (PCA) was also conducted. All statistical analyses were carried out using the JMP v. 18 software.

## 5. Conclusions

The results of this research indicate that the bioactive compounds present in ABJ, primarily phenolic acids and flavonoids, promote cytotoxicity, mild mitochondrial membrane depolarization, and reactive oxygen species (ROS) generation in SW480 and SW620 cells. Andean berry juice induced cell arrest in the S and G2/M phases, respectively, in SW480 and SW620 cells. MitoTracker analysis confirmed the cell cycle distribution results. Proteomic analysis of apoptosis-related markers further revealed a predominance of the extrinsic pro-apoptotic pathway in SW480 cells and of the intrinsic mitochondrial pathway in SW620 cells. More research is needed to validate these results using in vivo or clinical trials.

## Data Availability

Data will be available upon reasonable request.

## Supplementary Materials

The following supporting information can be downloaded at: https://www.mdpi.com/article/10.3390/molecules31122147/s1.

## Abbreviations

The following abbreviations are used in this manuscript:

ΔΨm	Mitochondrial membrane potential
ABJ	Andean Berry (*Vaccinium meridionale* Swartz) juice
ACE	Absolute cloning efficiency
ATCC	American Type Culture collection
Bad	Bcl-2-associated death promoter
Bax	Bcl-2 associated X protein
BCA	Bicinchoninic acid
Bcl-2	B-cell lymphoma 2
Bcl-XL	B-cell lymphoma-extra-large
CI	Confidence Interval
cIAP-1	Cellular inhibitor of apoptosis protein 1
cIAP-2	Cellular inhibitor or apoptosis protein 2
CDKN1A	Cyclin-dependent kinase inhibitor 1A
CRC	Colorectal cancer
Cyt. C	Cytochrome C
DiOC6	3,3-dihexylocarbocyanine iodide
DMEM	Dulbecco’s Modified Eagle’s Medium
DPPH	2,2-diphenyl-1-picrylhydrazyl
DR4	Death receptor 4
DR5	Death receptor 5
EGFR	Endothelial growth factor receptor
FADD	Fas-associated death domain protein
Fas (CD95)	FS-7-associated protein
FRAP	Ferric reducing antioxidant power
FBS	Fetal bovine serum
FDR	False discovery rate
HIF1a	Hypoxia-inducible factor 1, alpha subunit
HMOX2	Heme oxygenase 2
HPLC-DAD	High-performance liquid chromatography coupled to diode-array detection
HSP27	Heat-shock protein 27
HSP32	Heat-shock protein 32
HSP60	Heat-shock protein 60
HSP70	Heat-shock protein 70
HTRA	High-temperature requirement A
IC_50_	Half-inhibitory concentration
KEGG	Kyoto Encyclopedia of Genes and Genomes
KRAS	Kirsten rat sarcoma viral oncogene homolog protein
NF-κB	Nuclear factor kappa B
PI	Propidium iodide
PCA	Principal component analysis
PON2	Paraoxonase 2
RCE	Relative cloning efficiency
ROS	Reactive oxygen species
SMAC	Second mitochondria-derived activator of caspase
SRB	Sulforhodamine B
SSC	Side scatter
SW480	Human early colon cancer cells
SW620	Human metastatic colon cancer cells
TRAIL	Tumor necrosis factor-related apoptosis-inducing ligand
TNFRSF1A	Tumor necrosis factor receptor superfamily, member 1A
VEGFR	Vascular endothelial growth factor receptor
SMAC	Second mitochondria-derived activator of caspase
XIAP	X-linked inhibitor of apoptosis protein
