# Supplementary material for: Andean Berry (Vaccinium meridionale Swartz) Juice Promotes Cytotoxic and Proapoptotic Effects in Human Early-Stage and Metastatic Colorectal Cancer Cells
Source: Molecules. 2026 Jun 18;31(12):2147. doi: 10.3390/molecules31122147 (PMC13306007; doi:10.3390/molecules31122147)
Supplement: Supplementary file 1 [file molecules-31-02147-s001.zip › molecules-4226927-supplementary.pdf]

molecules

Supplementary Materials

Andean Berry (*Vaccinium meridionale* Swartz) Juice Promotes Cytotoxic and Proapoptotic Effects in Human Early-Stage and Metastatic Colorectal Cancer Cells

Ivan Luzardo-Ocampo [1,2], Myriam Agudelo-Quintero [3], Sandra S. Arango-Varela [4,5,*], Silvia A. Quijano [6,*], Maria E. Maldonado-Celis [7] and Jorge A. Lopera-Rodríguez [4,5]

1 Tecnologico de Monterrey, Institute for Obesity Research, Ave. Eugenio Garza Sada 2501 Sur., Col., Tecnológico, Monterrey 64700, Nuevo Leon, Mexico; ivanluzardo@tec.mx
2 Tecnologico de Monterrey, School of Engineering and Sciences, Ave. Eugenio Garza Sada 2501 Sur., Col., Tecnológico, Monterrey 64700, Nuevo Leon, Mexico
3 Research and Graduate Program in Food Science, School of Chemistry, Universidad Autónoma de Querétaro, Cerro de las Campanas S/N, Querétaro 76000, Queretaro, Mexico; magudelo07@alumnos.uaq.mx
4 Grupo de Investigación e Innovación Biomédica, Facultad de Ciencias Exactas y Aplicadas, Instituto Tecnológico Metropolitano (ITM), Calle 73 No. 76A-354 Vía al Volador, Medellín 050012, Antioquia, Colombia; alejandrolopera@itm.edu.co
5 Grupo de Investigación Biología Médica, Facultad de Ciencias Exactas y Aplicadas, Instituto Tecnológico Metropolitano (ITM), Calle 73 No. 76A-354 Vía al Volador, Medellín 050012, Antioquia, Colombia
6 Grupo de Investigación en Ecología y Conservación de la Biodiversidad (EcoBio), Facultad de Ciencias Básicas, Universidad Santiago de Cali, Cali 760001, Valle del Cauca, Colombia
7 Escuela de Nutrición y Dietética, Universidad de Antioquia, Carrera 75 No. 65-87, Medellín 050034, Antioquia, Colombia; maria.maldonado@udea.edu.co
* Correspondence: sandraarango@itm.edu.co (S.S.A.-V.); silvia.quijano00@usc.edu.co (S.A.Q.)

Table S1. Absolute cloning efficiency (ACE) and relative cloning efficiency (RCE) of SW480 and SW620 cells under several Andean berry (*Vaccinium meridionale* Swartz) (ABJ) treatments.

Treatment		ACE (%)	RCE (%)
SW480			
Untreated cells		25.5 ± 2.26	100
ABJ (mg/mL)	6	0	0
	12	0	0
	18	0	0
SW620			
Untreated cells		36.40 ± 8.48	100
ABJ (mg/mL)	6	0	0
	12	0	0
	18	0	0

The results are expressed as the mean ± S.D. of three independent experiments in triplicate. No statistical analysis was conducted because only untreated cells exhibited ACE and RCE values. ABJ: Andean berry (*Vaccinium meridionale* Swartz) juice; ACE: absolute cloning efficiency; RCE: relative cloning efficiency.

Table S2. Impact of the half-inhibitory concentration of Andean berry (*Vaccinium meridionale* Swartz) juice (ABJ) in the modulation of proteins related to the apoptotic process in SW480 and SW620 cells.

Protein Group	Protein	Protein (relative fold-change)	
		SW480	SW620
Proapoptotic	SMAC	-0.33 ± 0.03 [b]	-0.08 ± 0.01 [a]
	DR4	0.76 ± 0.48 [a]	-0.26 ± 0.04 [b]
	DR5	0.87 ± 0.28 [a]	-0.08 ± 0.00 [b]
	Bax	0.26 ± 0.43 [a]	-0.12 ± 0.02 [b]
	Bad	-0.19 ± 0.06 [a]	-0.08 ± 0.03 [b]
	Phospho-p53 (S15)	0.38 ± 0.07 [a]	-0.44 ± 0.01 [b]
	Phospho-p53 (S46)	0.25 ± 0.17 [a]	-0.28 ± 0.06 [b]
	Phospho-p53 (S392)	-0.21 ± 0.03 [a]	-0.27 ± 0.03 [a]
	Cytochrome c	-0.48 ± 0.03 [b]	0.13 ± 0.02 [a]
	Caspase-3	0.23 ± 0.20 [a]	-0.34 ± 0.01 [b]
	Proscaspase-3	-0.10 ± 0.13 [b]	0.36 ± 0.00 [a]
	Phospho-Rad17(S365)	-0.24 ± 0.05 [a]	-0.35 ± 0.08 [a]
Antiapoptotic	BCl-2	-0.32 ± 0.13 [a]	-0.41 ± 0.07 [a]
	Bcl-XL	1.63 ± 1.17 [a]	-0.18 ± 0.07 [b]
	XIAP	-0.23 ± 0.11 [a]	-0.53 ± 0.02 [a]
	Survivin	0.80 ± 1.22 [a]	-0.48 ± 0.01 [a]
	cIAP1	-0.39 ± 0.12 [a]	-0.45 ± 0.06 [a]
	cIAP2	-0.12 ± 0.10 [a]	-0.19 ± 0.03 [a]
	Livin	-0.49 ± 0.22 [a]	0.11 ± 0.06 [a]
Regulatory and stress response	Claspin	1.80 ± 1.08 [a]	-0.46 ± 0.10 [a]
	Fas	0.01 ± 0.06 [a]	-0.40 ± 0.05 [b]
	FADD	-0.03 ± 0.06 [a]	-0.19 ± 0.00 [b]
	Catalase	0.22 ± 0.17 [a]	0.30 ± 0.00 [a]
	HTRA2	-0.05 ± 0.10 [a]	-0.08 ± 0.03 [a]
	HSP27	1.03 ± 0.03 [a]	-0.09 ± 0.02 [b]
	HSP70	-0.30 ± 0.09 [b]	0.18 ± 0.05 [a]
	HSP32	-0.28 ± 0.09 [b]	0.33 ± 0.11 [a]
	HSP60	0.01 ± 0.05 [a]	0.02 ± 0.00 [a]
	HMOX2	-0.34 ± 0.00 [a]	-0.22 ± 0.08 [a]
	HIF1a	-0.01 ± 0.03 [b]	0.02 ± 0.00 [a]
	Clusterin	0.15 ± 0.43 [a]	-0.40 ± 0.09 [a]
	TNFRSF1A	-0.07 ± 0.21 [a]	-0.32 ± 0.01 [a]
	PON2	0.94 ± 0.04 [a]	-0.01 ± 0.01 [b]
	p21	0.50 ± 0.10 [a]	0.07 ± 0.04 [b]
	p27	0.17 ± 0.03 [a]	0.08 ± 0.04 [b]

The results are expressed as the mean ± S.D. of three independent experiments in triplicate. Different letters by row indicate significant differences ($p<0.05$) by Student's t-test. ABJ: Andean berry (*Vaccinium meridionale* Swartz) juice; Bad: Bcl-2-associated death promoter; Bax: Bcl-2-associated X protein; BCl-2: B-cell lymphoma 2; cIAP1: Cellular inhibitor of apoptosis protein 1; cIAP2: Cellular inhibitor of apoptosis protein 2; DR4: Death receptor 4; FADD: Fas-associated death domain protein; Fas (CD95): FS-7 associated protein; HIF1a: Hypoxia-inducible factor 1, alpha subunit; HMOX2: Heme oxygenase 2; HSP27: Heat-shock protein 27; HSP32: heat-shock protein 32; HSP60: Heat-shock protein 60; HSP70: Heat-shock protein 70; PON2: Paraoxonase 2; SMAC: Second mitochondria-derived activator of caspase; SW480: Human early-stage colon cancer cells; SW620: Human metastatic colon cancer cells; TNFRSF1A: Tumor necrosis factor receptor superfamily, member 1A; XIAP: X-linked inhibitor of apoptosis protein.

Table S3. Quantitative identification of hub nodes using degree centrality from the STRING Analysis of SW480 and SW620 cells

Protein	Node degree
CASP3	23
CYCS	23
BCL2	22
BCL2L1	22
DIABLO-2	19
XIAP	19
TNFRSF1A	18
FADD	17
TNFRSF10B	17
BAX	16
BIRC2	16
BIRC3	16
HSPA4	16
FAS	15
HIF1A	15
TNFRSF10A	15
HTRA2	14
BIRC7	12
HSPB1	12
BIRC5	11
HMOX1	10
CLU	9
HSPD1	7
BAD	6
HMOX2	2
CAT	1
CLSPN	1
PON2	1
TNFAIP8	1
DCTN6	0
TCEAL1	0

The results were obtained from STRING®, considering the fold-change values for each protein in IC_{50} ABJ-treated SW480 or SW630 cells. BAD: Bcl-2-associated death promoter; BAX: Bcl-2-associated X protein; BCL2: B-cell lymphoma 2; BCL2L1: Bcl-2-like protein 1; BIRC2: Baculoviral IAP repeat-containing protein 2; BIRC3 Baculoviral IAP repeat-containing protein 3 ; BIRC5: Baculoviral IAP repeat-containing protein 5; BIRC7: Baculoviral IAP repeat-containing protein 7; CASP3: Caspase 3; CAT: Catalase; CLSPN: Claspin; CLU: Clusterin; CYC2: Cytochrome C; DCTN6: Dynactin subunit 6; DIABLO-2: Correspond to the second mitochondria-derived activator of caspase; FADD: Fas-associated death domain protein; HIF1A: Hypoxia-inducible factor 1, alpha subunit; HMOX1: Heme oxygenase 1; HMOX2: Heme oxygenase 2; HSPA4: Heat-shock protein family A member 4; HSPB1: Heat-shock protein beta 1; HSPD1: 60 KDa Heat-shock protein; HTRA2: Serine protease; PON2: Paraoxonase 2; TCEAL1: Transcription elongation factor A protein-like 1; TNFAIP8: Tumor necrosis factor alpha-induced protein 8; TNFRSF10A: Tumor necrosis factor receptor superfamily, member 10A; TNFRSF10B: Tumor necrosis factor receptor superfamily, member 1A; TNFRSF1A: Tumor necrosis factor receptor superfamily, member 10B; XIAP: X-linked inhibitor of apoptosis protein.

Table S4. False discovery rate (FDR) values according to Benjamini-Hochberg adjusted p-values generated by STRING for the protein modulation in SW480 cells by the half-inhibitory concentration of Andean berry (*Vaccinium meridionale* Swartz) juice (IC_{50}-ABJ)

Term description	FDR
Biological process (gene ontology) enrichment	
Apoptotic process	4.42×10^{-19}
Regulation of apoptotic process	5.74×10^{-18}
Apoptotic signaling pathway	9.36×10^{-16}
Regulation of cysteine-type endopeptidase activity	9.36×10^{-16}
Negative regulation of apoptotic process	4.99×10^{-15}
Regulation of cysteine-type endopeptidase activity involved in apoptotic process	6.73×10^{-15}
Intrinsic apoptotic signaling pathway	3.18×10^{-14}
Positive regulation of protein metabolic process	2.18×10^{-12}
Extrinsic apoptotic signaling pathway	2.24×10^{-12}
Response to abiotic stimulus	3.15×10^{-12}
KEGG Pathways enrichment	
Apoptosis - multiple species	2.55×10^{-25}
Platinum drug resistance	2.04×10^{-21}
Apoptosis	1.92×10^{-14}
p53 signaling pathway	1.82×10^{-14}
Toxoplasmosis	1.76×10^{-14}
Small cell lung cancer	1.74×10^{-14}
Necroptosis	1.67×10^{-14}
Colorectal cancer	1.59×10^{-6}
NF-kappa B signaling pathway	1.59×10^{-6}
Measles	1.46×10^{-6}

Table S5. False discovery rate (FDR) values according to Benjamini-Hochberg adjusted p-values generated by STRING for the protein modulation in SW620 cells by the half-inhibitory concentration of Andean berry (*Vaccinium meridionale* Swartz) juice (IC_{50}-ABJ)

Term description	FDR
Biological process (gene ontology) enrichment	
Apoptotic process	4.42×10^{-19}
Regulation of apoptotic process	5.74×10^{-18}
Apoptotic signaling pathway	9.36×10^{-16}
Regulation of cysteine-type endopeptidase activity	9.36×10^{-16}
Negative regulation of apoptotic process	4.99×10^{-15}
Regulation of cysteine-type endopeptidase activity involved in apoptotic process	6.73×10^{-15}
Intrinsic apoptotic signaling pathway	3.18×10^{-14}
Positive regulation of protein metabolic process	2.18×10^{-12}
Extrinsic apoptotic signaling pathway	2.24×10^{-12}
Response to abiotic stimulus	3.15×10^{-12}
KEGG Pathways enrichment	
Apoptosis - multiple species	8.75×10^{-25}
Apoptosis	6.39×10^{-24}
Platinum drug resistance	5.44×10^{-19}
Toxoplasmosis	3.92×10^{-12}
Small cell lung cancer	3.74×10^{-12}
p53 signaling pathway	3.57×10^{-12}
Necroptosis	3.22×10^{-12}
Salmonella infection	2.89×10^{-9}
Measles	2.76×10^{-9}
Herpes simplex virus 1 infection	2.44×10^{-9}

Table S6. Participation of variables for each component of the PCA analysis.

Variable	PC1	PC2	PC3	PC4	PC5
Met. Act.	0.01334	0.24945	0.25500	0.15833	0.30556
Ki67	0.00079	-0.25548	0.13108	0.06900	-0.21516
Granularity	0.00233	0.25699	-0.05699	-0.01758	0.16386
SubG1	-0.00133	0.25494	-0.14862	-0.05670	0.11859
G0/G1	-0.00157	-0.25642	0.09239	-0.03311	0.08484
S	0.00173	0.25683	-0.06942	-0.03598	0.20488
G2/M	-0.00255	-0.25701	0.05498	0.00079	-0.10813
Q1	0.03595	0.16455	0.80717	0.35214	0.09919
Q2	0.00167	0.25652	-0.08727	0.02449	-0.04457
Q3	-0.00385	-0.25704	0.04763	-0.11406	0.25635
Q4	0.00631	0.25698	-0.02605	0.30788	-0.26736
MitoTracker	-0.00938	-0.23767	-0.40071	0.82413	0.27822
SMAC	0.18242	-0.00311	-0.00647	-0.00192	-0.00000
DR4	0.18242	-0.00311	-0.00647	-0.00192	-0.00000
Bax	-0.18242	0.00311	0.00647	0.00192	0.00000
Bad	-0.18242	0.00311	0.00647	0.00192	0.00000
pp53-S15	0.18242	-0.00311	-0.00647	-0.00192	-0.00000
pp53-S392	0.18242	-0.00311	-0.00647	-0.00192	-0.00000
Cyt. C	0.18242	-0.00311	-0.00647	-0.00192	-0.00000
Caspase 3	0.18242	-0.00311	-0.00647	-0.00192	-0.00000
Procaspase 3	-0.18242	0.00311	0.00647	0.00192	0.00000
pRAD17-S365	0.18242	-0.00311	-0.00647	-0.00192	-0.00000
Bcl-2	-0.18242	0.00311	0.00647	0.00192	0.00000
XIAP	-0.18242	0.00311	0.00647	0.00192	0.00000
Survivin	-0.18242	0.00311	0.00647	0.00192	0.00000
cIAP1	0.18242	-0.00311	-0.00647	-0.00192	-0.00000
cIAP2	0.18242	-0.00311	-0.00647	-0.00192	-0.00000
Claspin	-0.18242	0.00311	0.00647	0.00192	0.00000
Fas (CD95)	-0.18242	0.00311	0.00647	0.00192	0.00000
FADD	0.18242	-0.00311	-0.00647	-0.00192	-0.00000
Catalase	0.18242	-0.00311	-0.00647	-0.00192	-0.00000
HSP27	-0.18242	0.00311	0.00647	0.00192	0.00000
HSP70	-0.18242	0.00311	0.00647	0.00192	0.00000
HSP32	0.18242	-0.00311	-0.00647	-0.00192	-0.00000
HSP60	0.18242	-0.00311	-0.00647	-0.00192	-0.00000
HMOX2	0.18242	-0.00311	-0.00647	-0.00192	-0.00000
HIF1a	0.18242	-0.00311	-0.00647	-0.00192	-0.00000
Clusterin	-0.18242	0.00311	0.00647	0.00192	0.00000
TNFRSF1A	-0.18242	0.00311	0.00647	0.00192	0.00000
PON2	0.18242	-0.00311	-0.00647	-0.00192	-0.00000
p21	0.18242	-0.00311	-0.00647	-0.00192	-0.00000
p27	0.18242	-0.00311	-0.00647	-0.00192	-0.00000
D-Q1	-0.00303	0.25396	-0.17477	-0.12950	0.34597
D-Q2	0.00168	0.25681	-0.07056	-0.03792	0.20960
D-Q3	0.00296	0.25644	-0.08835	0.16589	-0.54599
D-Q4	-0.00198	-0.25638	0.09405	-0.08259	0.26535

Bad: Bcl-2-associated death promoter; Bax: Bcl-2-associated X protein; BCl-2: B-cell lymphoma 2; cIAP1: Cellular inhibitor of apoptosis protein 1; cIAP2: Cellular inhibitor of apoptosis protein 2; Cyt. C: Cytochrome C; DR4: Death receptor 4; FADD: Fas-associated death domain protein; Fas (CD95): FS-7 associated protein; HIF1a: Hypoxia-inducible factor 1, alpha subunit; HMOX2: Heme

oxygenase 2; HSP27: Heat-shock protein 27; HSP32: heat-shock protein 32; HSP60: Heat-shock protein 60; HSP70: Heat-shock protein 70; PON2: Paraoxonase 2; SMAC: Second mitochondria-derived activator of caspase; SW480: Human early-stage colon cancer cells; SW620: Human metastatic colon cancer cells; TNFRSF1A: Tumor necrosis factor receptor superfamily, member 1A; XIAP: X-linked inhibitor of apoptosis protein. Q1-Q4: Quadrants from the Annexin-V analysis; DQ1-DQ2: Quadrants from the DiOC-6 analysis.

Table S7. Binding affinities observed between selected polyphenolic compounds in Andean berry (*Vaccinium meridionale* Swartz) juice and protein targets from the apoptosis modulation in SW480 and SW620 cells.

Variable	Chlorogenic acid	Caffeic acid	Cyanidin-3-glucoside	Cyanidin-3-galactoside	Delphinidin-3-glucoside	Quercetin-3-glucoside hexoside
Bcl-2	-6.33 ± 0.21	-6.00 ± 0.17	-7.53 ± 0.06	-7.63 ± 0.21	-7.40 ± 0.00	-7.00 ± 0.20
Bcl-xL	-7.60 ± 0.10	-7.30 ± 0.20	-8.90 ± 0.00	-9.37 ± 0.60	-9.40 ± 0.78	-9.00 ± 0.17
Catalase	-7.63 ± 1.19	-7.67 ± 0.40	-9.27 ± 0.15	-9.07 ± 0.15	-9.60 ± 0.17	-10.30 ± 0.95
cIAP-2	-6.50 ± 0.00	-5.30 ± 0.00	-6.77 ± 0.29	-6.87 ± 0.15	-7.20 ± 0.00	-7.07 ± 0.06
Cytochrome C	-6.73 ± 0.23	-6.40 ± 0.35	-7.17 ± 0.21	-7.40 ± 0.26	-7.67 ± 0.23	-7.63 ± 0.21
Claspin	-7.33 ± 0.15	-6.60 ± 0.00	-9.23 ± 0.06	-9.10 ± 0.00	-9.27 ± 1.10	-8.50 ± 0.00
Fas	-7.53 ± 0.35	-6.23 ± 0.12	-8.20 ± 0.26	-8.23 ± 0.59	-8.77 ± 0.51	-8.67 ± 0.23
Hsp27	-8.90 ± 0.10	-7.20 ± 0.10	-8.60 ± 0.00	-9.70 ± 0.00	-9.03 ± 0.55	-9.83 ± 0.25
Hsp60	-6.17 ± 0.15	-5.27 ± 0.06	-7.00 ± 0.10	-7.13 ± 0.46	-7.43 ± 0.23	-6.90 ± 0.10
PON-2	-8.53 ± 0.15	-6.30 ± 0.00	-9.47 ± 0.58	-9.50 ± 0.00	-9.67 ± 0.06	-9.40 ± 0.00
Procaspase 3	-6.67 ± 0.06	-5.43 ± 0.06	-7.43 ± 0.12	-7.63 ± 0.12	-8.23 ± 0.06	-8.47 ± 0.06
DR4	-8.10 ± 0.30	-6.00 ± 0.00	-9.07 ± 0.15	-8.60 ± 0.20	-9.17 ± 0.35	-9.73 ± 0.06
DR5	-7.47 ± 0.12	-5.97 ± 0.06	-8.57 ± 0.06	-8.37 ± 0.15	-9.13 ± 0.12	-8.37 ± 0.06
XIAP	-6.47 ± 0.06	-5.07 ± 0.06	-6.63 ± 0.25	-6.57 ± 0.06	-6.93 ± 0.12	-7.40 ± 0.17

The results are expressed as the mean ± S.D. of three independent simulations. Simulations were conducted by downloading each polyphenolic compound from PubChem and each protein target from the Protein Data Bank, preparing the receptors and ligands in Biovia Discovery Studio, and docking them using AutoDock Tools. Bad: Bcl-2-associated death promoter; Bax: Bcl-2-associated X protein; BCl-2: B-cell lymphoma 2; Bcl-XL: B-cell lymphoma-extra-large; cIAP-2: Cellular inhibitor of apoptosis protein 2; DR4: Death receptor 4; DR5: Death receptor 5; Fas: FS-7-associated protein; Hsp27: Heat-shock protein 27; Hsp60: Heat-shock protein 60; PON2: Paraoxonase 2; XIAP: X-linked inhibitor of apoptosis.

Table S8. Chemical standards (HPLC-grade) identified in Andean berry (*Vaccinium meridionale* Swartz) juice.

Compound Classes	Chemical compound	Content (µg/mL)	λ (nm)	Retention time (min)	Standard curve and regression coefficient (R^2)
Phenolic compounds (µg/g)	Gallic acid	389.92 ± 27.84	280	1.856	y = 29814x + 125.69 (R^2: 0.9996)
	Chlorogenic acid	752.00 ± 22.96	280	3.311	y = 56690x - 208.28 (R^2: 1.000)
	Caffeic acid	172.81 ± 5.00	280	4.586	y = 75693x + 382.11 (R^2: 0.9995)
	Ellagic acid	410.64 ± 2.49	280	5.567	y = 10950x + 2591 (R^2: 0.9824)
	Vanillin	21.08 ± 0.75	280	6.043	y = 119632x – 1057.9 (R^2: 0.9994)
	Sinapic acid	81.96 ± 0.89	280	6.284	y = 33987x + 312.41 (R^2: 0.9992)
	Rutin	100.87 ± 2.35	320	5.020	y = 18144x – 36.546 (R^2: 0.9999)
	Quercetin	37.94 ± 3.45	320	12.121	y = 45262x – 565.14 (R^2: 0.9992)
	Kaempferol	2.54 ± 0.11	320	13.325	y = 2535x – 14.503 (R^2: 0.9884)
Oligosaccharides (mg/g)	Raffinose	0.78 ± 0.10	-	3.012	y = 147996x + 4545.3 (R^2: 0.9804)
	Verbascose	0.72 ± 0.12	-	5.760	y = 786102x + 44829 (R^2: 0.9910)
	Stachyose	0.51 ± 0.09	-	7.690	y = 327966x – 5998.5 (R^2: 0.9968)

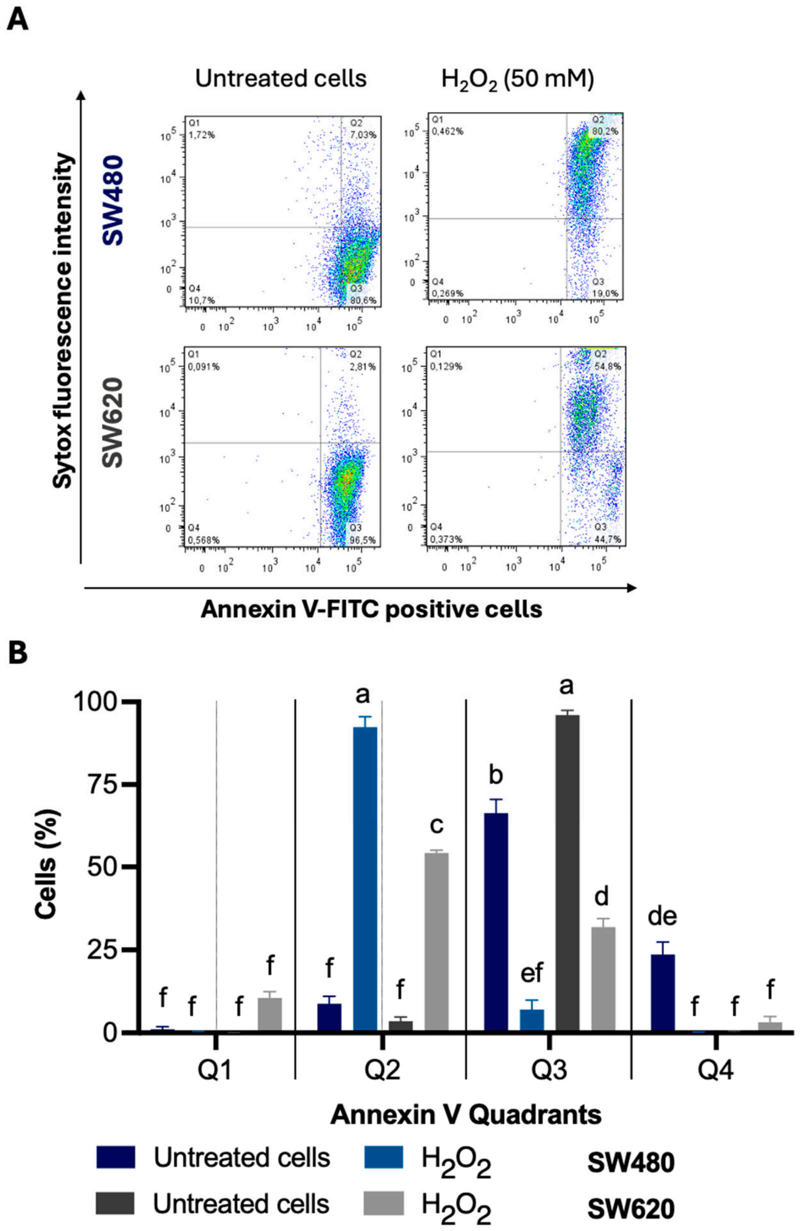

Figure S1. Mitochondrial membrane potential (ΔΨm) representative flow cytometry pictures of untreated cells (negative control) and H_2O_2-treated (positive control) SW480 and SW620 cells.

The results were expressed as the mean ± S.D. of at least two independent experiments in triplicate. Different letters express significant differences ($p<0.05$) by Tukey-Kramer's test. Untreated cells corresponded to either SW480 or SW620 cells in 2% FBS-DMEM. Q1 (DiOC6−/PI+): dying cells (nonapoptotic/necrotic), low ΔΨm, and low membrane integrity; Q2 (DiOC6+/PI−): Late apoptosis, high ΔΨm, and low membrane integrity; Q3 (DiOC6+/PI−): High ΔΨm and proper membrane integrity; Q4 (DiOC6−/PI−): low ΔΨm, and good membrane integrity.

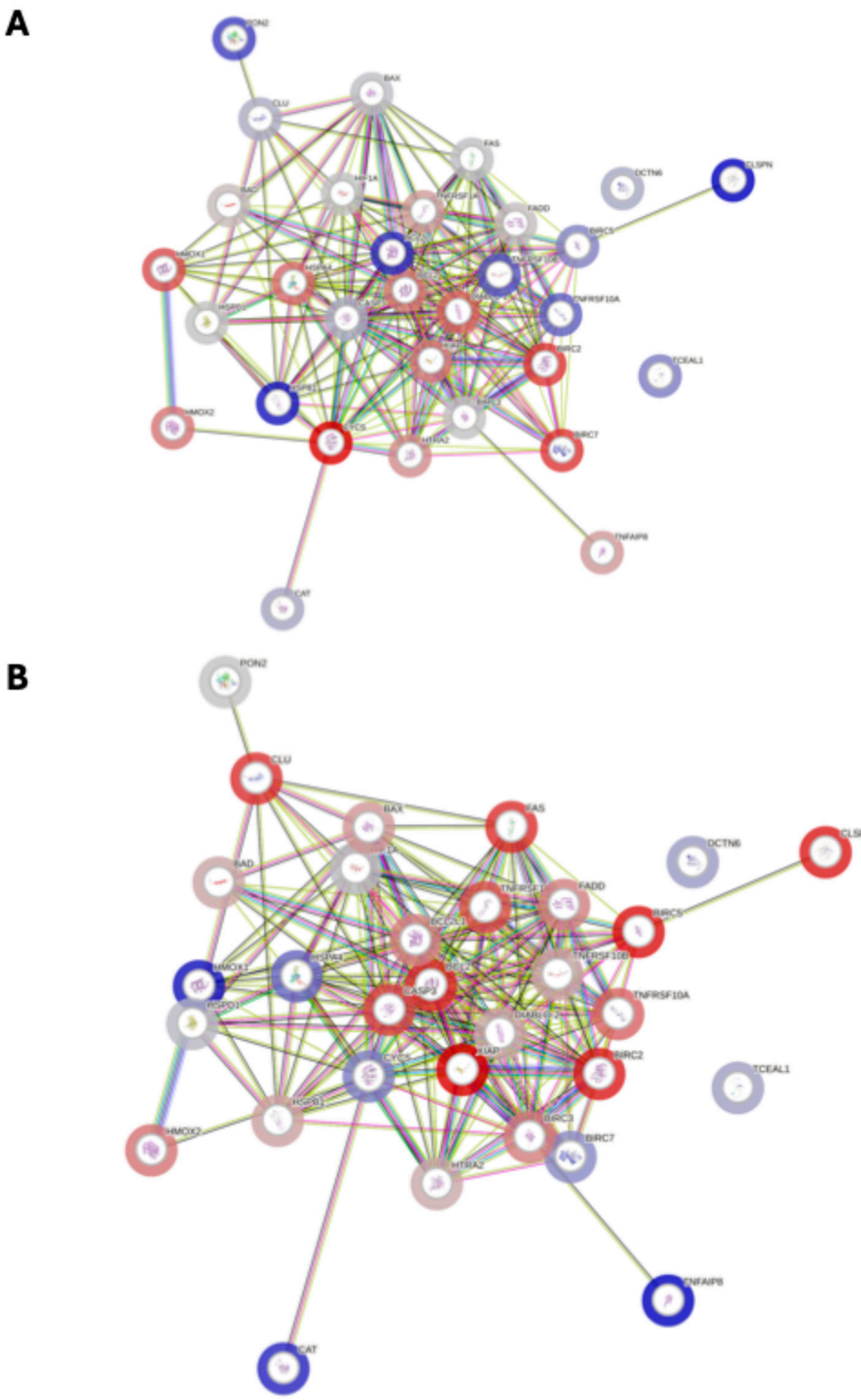

Supplementary Figure S2. Protein network from the STRING® analysis of the impact of the half-inhibitory concentration of Andean berry (*Vaccinium meridionale* Swartz) juice (IC_{50} ABJ) in **(A)** SW480 and **(B)** SW620 cells. The results were obtained from STRING®, considering the fold-change values for each protein in IC_{50} ABJ-treated SW480 or SW630 cells. BAD: Bcl-2-associated death promoter; BAX: Bcl-2-associated X protein; BCL2: B-cell lymphoma 2; BCL2L1: Bcl-2-like protein 1; BIRC2: Baculoviral IAP repeat-containing protein 2; BIRC3 Baculoviral IAP repeat-containing protein 3 ; BIRC5: Baculoviral IAP repeat-containing protein 5; BIRC7: Baculoviral IAP repeat-containing protein 7; CASP3: Caspase 3; CAT: Catalase; CLSPN: Claspin; CLU: Clusterin; CYC2: Cytochrome C; DCTN6: Dynactin subunit 6; DIABLO-2: Correspond to the second mitochondria-derived activator of caspase; FADD: Fas-associated death domain protein; HIF1A: Hypoxia-inducible factor

1, alpha subunit; HMOX1: Heme oxygenase 1; HMOX2: Heme oxygenase 2; HSPA4: Heat-shock protein family A member 4; HSPB1: Heat-shock protein beta 1; HSPD1: 60 KDa Heat-shock protein; HTRA2: Serine protease; PON2: Paraoxonase 2; TCEAL1: Transcription elongation factor A protein-like 1; TNFAIP8: Tumor necrosis factor alpha-induced protein 8; TNFRSF10A: Tumor necrosis factor receptor superfamily, member 10A; TNFRSF10B: Tumor necrosis factor receptor superfamily, member 1A; TNFRSF1A: Tumor necrosis factor receptor superfamily, member 10B; XIAP: X-linked inhibitor of apoptosis protein.

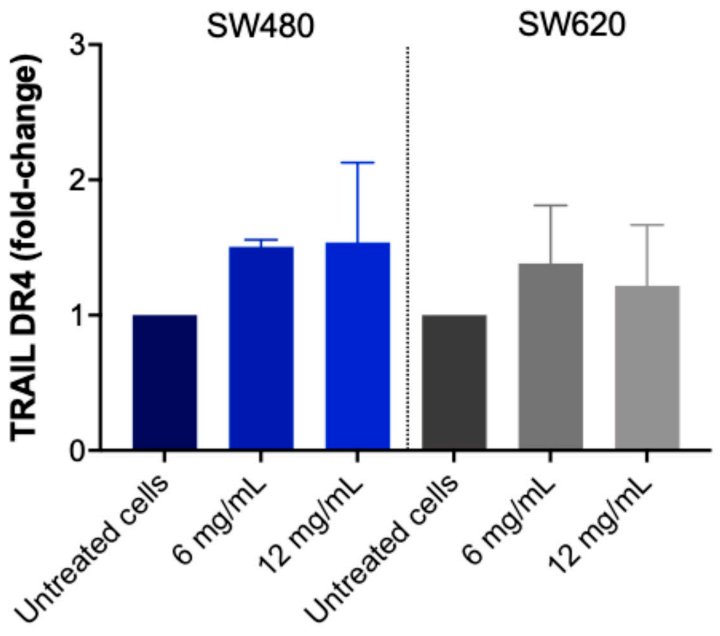

Figure S3. TRAIL-DR4 protein levels (in fold-change) in SW480 and SW620 cells without and with ABJ treatments (6 and 12 mg/mL). The results are expressed as the mean ± S.D. No differences ($p>0.05$) were shown between the treatments by Tukey-Kramer's test. ABJ: Andean berry (*Vaccinium meridionale* Swartz) juice; TRAIL DR4: Tumor necrosis factor (TNF)-related apoptosis-inducing ligand death receptor 4; SW480: Human early-stage colon cancer cells; SW620: Human metastatic colon cancer cells.

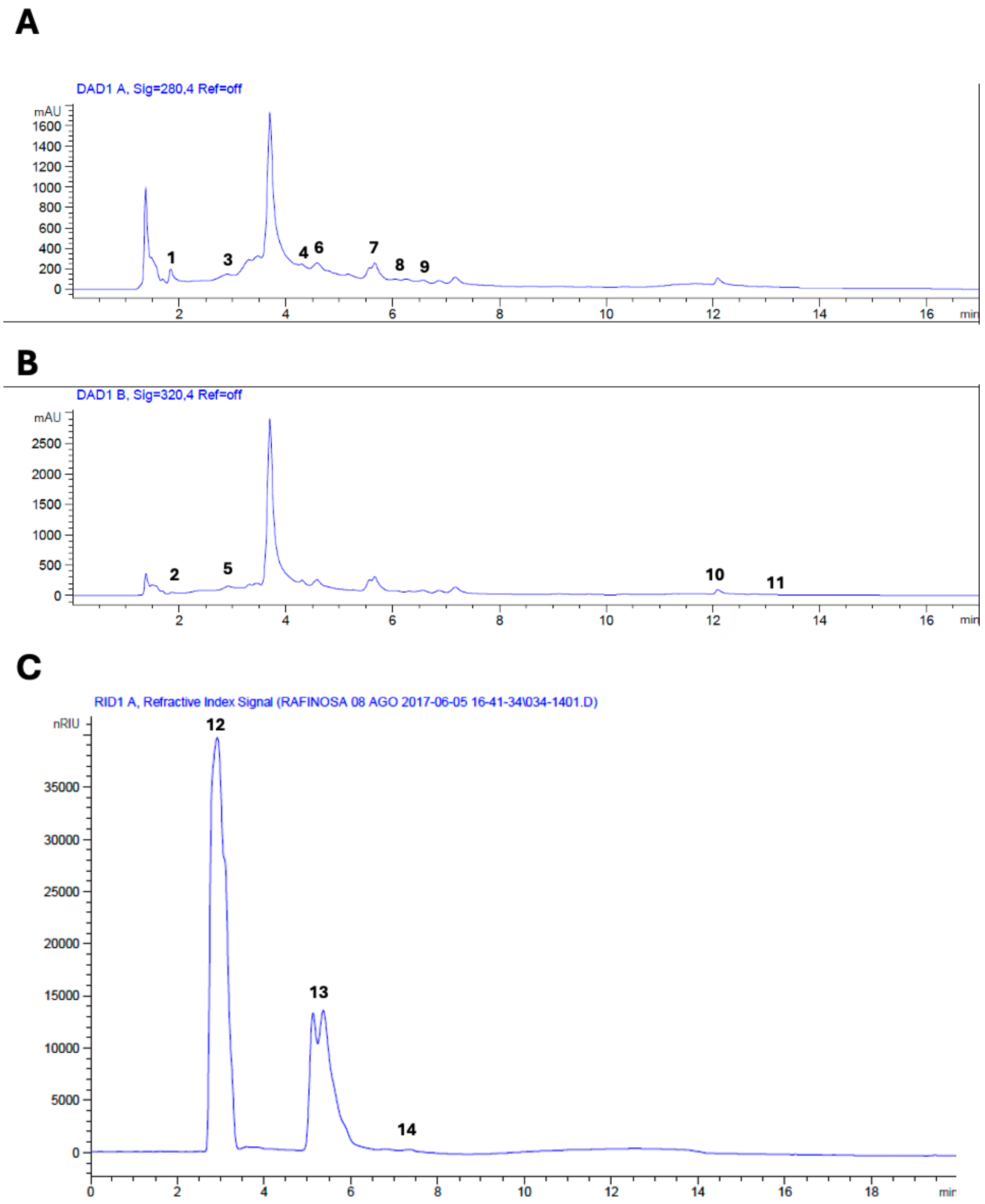

Figure S4. Representative chromatogram of the main phenolic compounds and oligosaccharides identified in the methanolic (80 % v/v) and aqueous extracts, respectively, of Andean berry (*Vaccinium meridionale* Swartz) juice, **(A)** Phenolic compounds identified at 280 nm by HPLC-DAD; **(B)** Phenolic compounds identified at 320 nm by HPLC-DAD; **(C)** Oligosaccharides identified by HPLC-RID

Identified compounds were as follows: **(1)** gallic acid; **(2)** (+)-catechin; **(3)** chlorogenic acid; **(4)** caffeic acid; **(5)** rutin; **(6)** *p*-coumaric acid; **(7)** ellagic acid; **(8)** Vanillin; **(9)** Sinapic acid; **(10**) Quercetin; **(11)** Kaempferol; **(12)** Raffinose; **(13)** Stachyose; **(14)** Verbascose.

Disclaimer/Publisher's Note: The statements, opinions and data contained in all publications are solely those of the individual author(s) and contributor(s) and not of MDPI and/or the editor(s). MDPI and/or the editor(s) disclaim responsibility for any injury to people or property resulting from any ideas, methods, instructions or products referred to in the content.
